# Eating Disorders and Disordered Eating Symptoms in Adolescents with Type 1 Diabetes

**DOI:** 10.3390/nu9080906

**Published:** 2017-08-19

**Authors:** Giada Toni, Maria Giulia Berioli, Laura Cerquiglini, Giulia Ceccarini, Ursula Grohmann, Nicola Principi, Susanna Esposito

**Affiliations:** 1 Pediatric Clinic, Università degli Studi di Perugia, Piazza Menghini 1, 06129 Perugia, Italy; toni.giada@gmail.com (G.T.); mgiuliaberioli@gmail.com (M.G.B.); laura.cerquiglini@ospedale.perugia.it (L.C.); giulia.cec@hotmail.it (G.C.); 2 Department of Experimental Medicine, Università degli Studi di Perugia, 06129 Perugia, Italy; ursula.grohmann@unipg.it; 3 Pediatric Highly Intensive Care Unit, Università degli Studi di Milano, Fondazione IRCCS Ca' Granda Ospedale Maggiore Policlinico, 20122 Milan, Italy; nicola.principi@unimi.it

**Keywords:** adolescents, disordered eating symptoms, eating disorders, type 1 diabetes

## Abstract

Eating problems in adolescents with type 1 diabetes (T1D) can be divided into two groups. The first includes the diagnosed eating disorders (EDs), i.e., diseases specifically identified by defined signs and symptoms for which a degree of severity has been established, such as anorexia nervosa, bulimia nervosa, binge-eating disorder, pica, and rumination. The second is the group of disordered eating symptoms (DES), which include behaviors such as dieting for weight loss, binge eating, self-induced vomiting, excessive exercise, and laxative or diuretic use; these behaviors cannot be categorized as complete diseases, and, although apparently mild, they must be closely evaluated because they can evolve into true EDs. In this review, present knowledge about the clinical relevance of EDs and DES and the possible preventive and therapeutic measures used to reduce their impact on the course of T1D will be discussed. As adolescents with diabetes are at higher risk of eating disturbances and consequently for higher rates of disease complications, care providers should pay attention to clinical warning signs that raise suspicion of disturbed eating to refer these patients early to an expert in nutrition and mental health disorders. To ensure the best care for adolescents with T1D, diabetes teams should be multidisciplinary and include a pediatric diabetologist, a skilled nurse, a dietician, and a psychologist.

## 1. Introduction

Type 1 diabetes (T1D) is a severe chronic disease that can be diagnosed at any age. However, most of the cases first develop in children and adolescents, with peaks in children 5–7 years old and near puberty [1,2]. The incidence and prevalence of T1D vary significantly from country to country, with the highest values in Finland and in Sardinia, Italy (>60 cases and >40 cases per 100,000 people each year, respectively), and the lowest in China, India, and Venezuela (approximately 0.1 cases per 100,000 people per year). However, T1D represents between 10% and 15% of all cases of diabetes that are diagnosed annually, and its incidence is increasing at 3% per year worldwide [3]. 

T1D occurs secondary to partial or total destruction of pancreatic β-cells, mainly due to autoimmunity [4]. This destruction causes chronic lack of insulin, emergence of hyperglycemia, and severe metabolic problems. Macrovascular and microvascular complications are common [3]. However, psychiatric disorders such as anxiety and depression can also occur, particularly in adolescents, and lead to disordered eating behaviors that can complicate the clinical course of T1D [5]. Eating problems in T1D patients can be divided in two groups [6,7]. The first includes the diagnosed eating disorders (EDs), i.e., diseases that are specifically identified by defined signs and symptoms for which a degree of severity has been established, such as anorexia nervosa (AN), bulimia nervosa (BN), binge-eating disorder, pica, and rumination. The second is the group of disordered eating symptoms (DES), which include behaviors such as dieting for weight loss, binge eating, self-induced vomiting, excessive exercise, and laxative or diuretic use; these types of behaviors cannot be categorized as complete diseases, and although apparently mild, they must be closely evaluated because they can evolve into true EDs. In this review, the current knowledge of the clinical relevance of EDs and DES as well as the preventive and therapeutic measures that can be used to reduce their impact on the course of T1D will be discussed.

## 2. Clinical Relevance of Unhealthy Eating Behaviors in Adolescents with Type 1 Diabetes (T1D)

Unhealthy eating behaviors, particularly EDs, are associated with worse metabolic control and higher rates of diabetes complications, including serious medical risks and premature mortality. Scheuing et al. evaluated the clinical characteristics and outcomes of 52,215 patients with T1D, 467 of whom had a clinically recognized ED [8]. Ketoacidosis and hospitalization were found to be significantly more common (*p* < 0.05), and the duration of hospital stay longer (*p* < 0.05) in patients with T1D with psychiatric problems than in those without. Interestingly, the risk of T1D-related clinical problems seemed strictly related to the type of unhealthy eating behavior. Patients with BN and binge-eating disorder had a 2.5-fold (95% confidence interval (CI): 1.3–4.8) and a 1.4-fold (95% CI: 0.8–2.3) higher risk of retinopathy, respectively, whereas AN patients had no increased risk (95% CI: 0.4–2.0). Moreover, the degree of severity of EDs could significantly influence the progression of T1D. In a study by Rydall et al., it was shown that diabetic retinopathy could be diagnosed four years after first control in 86% and 43% of patients with severe or moderate EDs and in 24% of those with non-disordered eating behavior [9]. 

Poor control of T1D due to psychiatric problems leads to severe T1D complications. Insulin omission has been associated with recurrent diabetic ketoacidosis events, with marked increases in mortality risk [10]. In a study conducted with women with T1D, AN or both diseases, after 10 years of follow-up, the crude mortality rates were 2.5%, 6.5%, and 34.8% for T1D, AN, and concurrent cases, respectively [11]. Disturbed eating behaviors are also associated with recurrent episodes of severe hypoglycemia, which can lead to heart complications, kidney failure, cerebral edema, coma, or death [12]. 

## 3. Prevalence of EDs and DES

Several studies have evaluated the prevalence of EDs and DES in adolescents with T1D [13,14,15,16,17,18,19,20,21]. With few exceptions, people with T1D were more prone to developing eating problems than healthy people. In a meta-analysis of five studies including 664 adolescents with and 1894 without T1D [13,15,18,19,20], Young et al. reported that DES could be detected in 39.3% of patients and in 32.5% of controls (effect size *d* = 0.52; 95% CI: 0.10-0.94) [22]. Similar differences were found in the same meta-analysis when the six studies on EDs were considered [13,14,16,17,18,20]. After data from 825 individuals with T1D and 2282 peers without T1D were grouped, ED was diagnosed in 7.0% of subjects with T1D and in only 2.8% of controls (*d* = 0.46; 95% CI: 0.10–0.81). However, if the risk of eating problems is likely to be significantly greater in adolescents with T1D than in those without, the true difference between patients and controls has not been precisely defined. 

The prevalence of EDs and DES is usually established through the administration of questionnaires that are not specifically designed for patients with T1D, and this methodology can lead to under- or over-estimation of eating problems. Questionnaires do not include questions regarding eating problems that are specific to T1D, such as insulin omission [23]. Furthermore, they tend to consider certain eating behaviors that are integral to diabetic care, such as attention to diet and refusal to eat some foods, as expressions of disease [24]. A second problem can arise from the characteristics of the studies planning to evaluate the prevalence of eating problems in individuals with T1D. The prevalence values have been shown to vary according to the different ED and DES categories used and the people studied. Mannucci et al. compared the prevalence of mental disorders in 748 and 1587 female subjects with and without T1D, respectively [25], and found that the prevalence of AN was quite similar in both groups (0.27% vs. 0.06%), whereas that of BN was significantly greater in T1D patients (1.7% vs. 0.7%; *p* < 0.05). However, when considering AN and BN together, the prevalence of psychiatric problems was higher in patients with T1D than in controls (2.0% vs. 0.75%, *p* < 0.05). Finally, the measure used to assess eating behaviors in the various studies has not been uniform, and these discrepancies can lead to different results. When measures are adapted to T1D, i.e., validated in a population with T1D or adapted to consider both insulin misuse and the restricted diet in T1D, the differences between T1D patients and controls are significantly lower than when generic measures are used. In a meta-analysis by Young et al., the prevalence of EDs in T1D patients was 10.1% and 6.4% when generic and diabetes-adapted measures, respectively, were used [22]. Because the prevalence remained at approximately 3% in controls, the difference generated by using diabetes-adapted measures was not significant (*d* = 0.43; 95% CI: −0.06–0.91).

## 4. Risk Factors for the Development of Eating Disorders (EDs) and Disordered Eating Symptoms (DES)

Several prospective studies have identified factors that increase the risk of developing psychiatric problems that lead to EDs and DES [26,27]. In individuals with T1D, age, sex, a dietetic approach to T1D, body mass index, body dissatisfaction, family support and T1D complications seem to play a major role in this increased risk (Table 1).

### 4.1. Age and Sex

As previously reported, more cases of T1D develop during adolescence. Adolescence is a critical period of human development during which several physical and psychological changes occur, and, unfortunately, some of them can favor unhealthy dietary behaviors [28]. Peer influences and continuous use of social media may increase the risk of developing eating problems. Takii et al. conducted a study to determine the age of T1D onset that was most closely related to the subsequent development of severe EDs [29]. They reported that individuals with T1D with an onset between the ages of 7 and 18 years were at a significantly higher risk of subsequently developing a severe ED, such as AN or BN, than subjects whose onset was either younger or older. Females are at greater risk than males. This difference by sex has been clearly demonstrated and seems independent of the presence of underlying disease. In general, among adolescents, EDs and DES considered together occur in 3.8% of females and in 1.5% of males [28]. In adolescents with T1D, these conditions can be detected in 37.9% of females and 15.9% of males [30]. Additionally, compliance with prescribed therapy for T1D was found to be significantly lower in females than in males, suggesting that females may experience more frequent psychiatric problems. Neumark-Sztainer et al. found that 10.3% of adolescent girls skipped insulin and that 7.4% took less insulin to lose weight, compared to only 1.4% of males [31]. Similar findings were reported by Wisting et al., who enrolled 105 adolescents with T1D in a study that investigated which factors were correlated with ED psychopathology [32]. They found that insulin restriction, together with age and illness perception, was relatively strongly associated with ED among females only, whereas no association was demonstrated among males.

### 4.2. Dietetic Approach to Type 1 Diabetes (T1D)

Detailed meal planning, precision in food portions and constant monitoring of carbohydrate intake are essential to the management of T1D but may place patients at higher levels of concern regarding health and food and can lead to the emergence of psychiatric problems that form the basis for ED and DES development [33].

In some cases, carbohydrate restriction was even greater than prescribed due to patients’ excessive concerns about food [14]. Moreover, some patients prefer to practice extreme dieting than exercise to control T1D [34]. The dietary restraints imposed by T1D management can lead to craving of forbidden foods and result in binging behavior, especially in secret from parents and at prohibited times. Patients are allowed to eat sweetened foods and drinks that are normally forbidden to correct for hypoglycemia, but they often eat too much to satisfy the intense hunger caused by hypoglycemic symptoms, without control or supervision. This behavior can create a vicious cycle because patients may feel guilty about consuming these foods and consequently restrict their eating, resulting in another hypoglycemic episode. This pattern of behavior is similar to the over-eating and guilt cycle of individuals with BN [35].

### 4.3. Body Mass Index, Body Dissatisfaction, and Family Support

In adolescents with T1D, large fluctuations in body weight usually occur. At onset of T1D, a reduction in body weight is common. In contrast, treatment with insulin frequently leads to an increase in body weight due to increased metabolic efficiency [36]. Ingberg et al. studied 18 adolescent females with T1D, 16–19 years of age, and compared them to age-matched healthy controls [37]. They found that diabetic patients had a higher body mass index (BMI) than controls (26.3 ± 2.6 kg/m^2^ vs. 23.6 ± 3.8 kg/m^2^; *p* < 0.05). The occurrence of overweight was almost entirely due to increased fat mass, which was mainly distributed in the upper part of the body. This distribution was significantly correlated to diabetes control, as indicated by the glycated hemoglobin (HbA1c) value (*r* = 0.69; *p* < 0.005). A clear relationship between fat distribution and both daily dosage of insulin (*r* = 0.78; *p* < 0.0005) and total cholesterol (*r* = 0.60; *p* < 0.001) was also observed. Overweight and obesity in T1D patients can lead to eating problems and poor compliance with therapy. Tse et al. examined 151 adolescents with T1D and found that those who were classified as at-risk for EDs according to their eating-related attitudes were more likely to be overweight/obese than those at low risk (59.1% vs. 31.8%; *p* = 0.01) [38]. Moreover, compared with low-risk individuals, at-risk individuals were less compliant with T1D therapy (*p* < 0.01), less frequently monitored their blood glucose concentration (*p* < 0.002), and had higher HbA1c values (*p* < 0.001). 

Body dissatisfaction is the main reason for the development of dieting, disordered eating and reduction or elimination of prescribed insulin dosages. Body image has long been shown to regulate quality and quantity of food intake [39]. Moreover, the evidence clearly indicates that pathology in self-regulative mechanisms due to undesired life events including overweight and obesity may lead to the development of a negative body image followed by unhealthy eating behaviors [40]. The importance of body weight in conditioning the development of EDs in adolescents with T1D was documented in a recent study by Gonçalves et al. [41], who assessed 79 subjects of both sexes with T1D using several validated questionnaires that were specifically designed to evaluate eating disorders [42], self-estimation [43], anxiety [44], and quality of life [45]. Patients with the desire to decrease their current weight were significantly more anxious than those who preferred to maintain or increase their weight (*p* < 0.001). Moreover, patients who wanted to lose weight showed decreased quality of life, mainly due to the negative impact of T1D and worries about the disease, and they exhibited more restraints and more eating, weight, and shape concerns. A significant association between desire to reduce weight and emergence of particular eating behaviors was observed. Self-induced vomiting and laxative use were reported in 48.6% of patients who wanted to lose weight compared to 2.3% and 11.4%, respectively, of those who did not. 

The psychological problems of adolescents with T1D can be worsened by their family eating and diabetes management environments. Poor attention to healthy eating by the family has been associated with a greater prevalence of disordered eating behaviors in adolescents with T1D [46]. Moreover, eating problems in adolescents with T1D are more common in the presence of maternal weight and shape concerns and when mothers have been involved in binge-eating disorders themselves [47]. Family support seems essential to reducing the risk of unhealthy eating behaviors [48], and weight-related teasing by parents can lead to a higher frequency of eating problems [49].

## 5. Screening Tools and Diagnosis

The diagnosis of EDs and DES in individuals with T1D is difficult due to the frequent concealment and denial of disordered eating behavior. Therefore, care providers must have a high index of suspicion and pay attention to warning clinical signs (Table 2). 

### 5.1. Signs of Suspicion

The first sign that should alert care providers to the possibility of EDs or DES is poor glycemic control. Mean HbA1c levels are usually higher in patients with eating problems than in those without these problems. Rydall et al. found that the mean HbA1c values were significantly higher (11.1% ± 1.2%) in the group with highly disordered eating behaviors than in the group with moderately disordered (8.9% ± 1.7%) or non-disordered eating behaviors (8.7% ± 1.6%) [9]. Similar results were reported by Wisting et al., who showed in a study of 770 11–19-year-old children and adolescents with T1D that those with eating problems had significantly higher HbA1c values (9.2% ± 1.6%) than those without (8.4% ± 1.3%; *p* < 0.001) [50]. Finally, using a computer model of data mining, Pinhas-Hamiel et al. showed that adolescent females with intentional insulin omission had poorer glycemic control and could be distinguished by HbA1c values >9.2% and by HbA1c measurements more than 20% above the 90th percentile [51].

Recurrence of hypoglycemic episodes is a second relevant index of suspicion for unhealthy earing behaviors. These episodes appear to be relatively common among T1D patients engaging in binge eating and self-induced vomiting. Increased self-administration of insulin has been performed to induce hypoglycemia and justify eating sweets and high carbohydrate meals. This type of behavior was described in 22.8% of 241 adolescents with T1D in a study by Schober et al. [52]. 

Systematic calculations of caloric values, weighing of foods, frequently missed medical check-ups, refusal to be weighed, concerns about appearance, and a tendency to favor vegetarianism are other characteristics and signs of possible EDs or DES as well as anxiety, mood, and personality and behavior disorders [27]. Screening for disordered eating should begin in pre-adolescence and continue through early adulthood to ensure that treatment is provided as early as possible.

Longitudinal studies of disordered eating behaviors in patients with T1D indicate that these behaviors are likely to persist and become more severe in young adulthood [27].

### 5.2. Diagnosis

The diagnosis of EDs is usually confirmed by questionnaires specifically designed to evaluate unhealthy eating behaviors in T1D patients. These assessments contain questions regarding eating, weight and self-estimates and are generally derived by removing questions related to diabetes-imposed dietary restrictions from questionnaires designed to identify patients with EDs or DES.

The revised Diabetes Eating Problem Survey (DEPS-R) includes 16 items. It can be completed in less than 10 minutes and has excellent internal consistency and specificity. In a study used to validate the DEPS-R, adolescents with a score greater than 1 standard deviation above the mean were at an increased risk of eating problems [23]. 

A second questionnaire used for identification of eating disorders is the Eating Disorder Inventory 3 (EDI-3) [53,54], which has been modified to be used in patients with T1D. The test was validated in a study enrolling 356 adolescent females with T1D and 1098 age-matched healthy controls [13]. Another test used to assess EDs in T1D patients is the modified SCOFF (mSCOFF), which is a simple five-item screening tool that can be easily implemented during a follow-up visit and has demonstrated reliability and validity [55]. This is a modified version, specifically developed for T1D patients, of the original SCOFF ED screening questionnaire. Practically, the last question of the SCOFF ED (“Would you say that food dominates your life?”) was replaced with a new question strictly related to the management of T1D (“Do you ever take less insulin than you should?”). In a study in which it was compared with the EDI [53], it was found to have a sensitivity of 80% (95% CI: 44–97) and a specificity of 90% (95% CI: 76–98) 

Finally, one study has investigated the use of a single question, “Have you ever been overweight?” to screen for the presence of disordered eating in adolescents with T1D. This one question yielded an 83% sensitivity and 94% negative predictive value, and thus it may be an excellent question for when time is limited to select individuals for whom deeper investigations are needed [56]. For adolescents, the presence of family members in the clinic visit may affect their honest responses to basic screening questions; therefore, it may be beneficial to ask the family member to step out for a moment.

## 6. Interventions for Prevention and Treatment

Studies suggest that disordered eating behaviors that start in adolescent years persist into adulthood, especially if left untreated. The results of these studies also suggest that EDs and DES do not usually resolve without treatment [27].

To ensure the best care for adolescents with T1D, diabetes teams should be multidisciplinary and consist of a pediatric diabetologist, a skilled nurse, a dietician, and a psychologist. Unfortunately, unhealthy eating behaviors often go untreated. According to some statistics, only 17% of girls and 1.8% of boys receive mental health services for eating disorders [57]. Table 3 summarizes the main useful interventions for adolescents with T1D and disordered eating behaviors. 

Interventions that aim to increase self-esteem and body acceptance and family-based interventions that aim to improve family management of diabetes might help reduce the risk of EDs in individuals with T1D [58]. Consultations and referrals to mental health services are an appropriate first step for screening and treatment. Various treatments exist for eating disorders, including family therapy, cognitive-behavioral therapy and interpersonal psychotherapy [59]. 

Low self-esteem, body dissatisfaction, and personality variables should be addressed in individual psychological treatment or group therapy. The concurrence of eating behavior disorders and mood disorders in patients with T1D supports the importance of treating depression as a means of treating EDs. If depression and anxiety are suspected, a psychiatric consultation is needed. As family dynamics may influence the development of EDs [25], family education may be an important component of treatment. More studies on the effects of family interventions as a treatment for adolescents with T1D and EDs and/or DES are needed. Nutritional counseling is also recommended as a component of successful therapy [12]. 

Finally, innovations in diabetes technology, such as continuous glucose monitoring (CGM) and insulin pumps (CSII), can have a relevant impact on eating behaviors [60]. Theoretically, they can have opposite effects. They can reduce EDs and DES clinical manifestations through the reestablishment of appetite and eating flexibility. On the contrary, they can make disordered eating worse because of an increased focus on caloric and carbohydrate intake. However, the use of modern technologies is associated with a significant decrease in insulin doses needed to maintain normal glucose levels. In a recent study carried out in adolescent with T1D, it was evidenced that use of CSII was associated with diminished endorsement of disordered eating behavior [60]. However, further studies are needed to evaluate the true impact of CGM and CSII on eating problems of T1D patients. 

## 7. Conclusions

As adolescents with diabetes are at a higher risk for eating disturbances and consequently for higher rates of disease complications, care providers should pay attention to warning clinical signs that raise suspicion of disturbed eating, as identification of at-risk patients can improve early referrals of these patients to experts in nutrition and mental health disorders. To ensure the best care for adolescents with T1D, diabetes teams should be multidisciplinary and include a pediatric diabetologist, a skilled nurse, a dietician, and a psychologist. 

## Figures and Tables

**Table 1:** Characteristics of eating disorders (EDs) and disordered eating symptoms (DES) among adolescents with type 1 diabetes.

Characteristic	Risk factor
Age	7–18 years
Gender	Female
Dietetic approach	Detailed meal planning, precision in food proportion
Body mass index	Overweight, obesity
Body perception	Body dissatisfaction
Personal characteristics	Anxious, poor quality of life
Family support	Poor attention in family to healthy eating, maternal overweight or binge-eating disorders in mothers

**Table 2:** Diagnosis of eating disorders (EDs) and disordered eating symptoms (DES) in adolescents with type 1 diabetes (T1D).

**Suspicion**
Poor glycemic control
Recurrence of hypoglycemic episodes
Systematic calculations of caloric values and weighing of foods
Frequently missed medical check-ups
Refusal to be weighed
Concern for appearance
Tendency toward vegetarianism
**Confirmation**
Revised Diabetes Eating Problem Survey (DEPS-R)
Modified SCOFF (mSCOFF) test
Single question: “Have you ever been overweight?”

**Table 3:** Main useful interventions for prevention and treatment of eating disorders (EDs) and disordered eating symptoms (DES) in adolescents with type 1 diabetes (T1D).

Intervention
Multidisciplinary approach with a pediatric diabetologist, a skilled nurse, a dietician, and a psychologist
Increased self-esteem and body acceptance
Family-based interventions
Nutritional counseling
Use of new technologies
